# Comparative Analysis of Meat Quality Characteristics of the *Longissimus dorsi* in Suffolk × Hu F_1_ Crossbreds and Their Parental Breeds

**DOI:** 10.3390/ani16071027

**Published:** 2026-03-27

**Authors:** Zhenghan Chen, Rui Zhang, Liwa Zhang, Zhenfei Xu, Xuejiao An, Chune Niu, Zhiguang Geng, Haina Shi, Jinxia Zhang, Lei Qu, Shuwei Dong, Yaojing Yue

**Affiliations:** 1 Shaanxi Provincial Engineering and Technology Research Center of Cashmere Goats, Life Science Research Center, Yulin University, Yulin 719000, China; 19139107648@163.com (Z.C.); ylqulei@126.com (L.Q.); 2 Key Laboratory of Animal Genetics and Breeding on the Tibetan Plateau, Ministry of Agriculture and Rural Affairs, Lanzhou Institute of Husbandry and Pharmaceutical Sciences, Chinese Academy of Agricultural Sciences, Lanzhou 730050, China; zhangrui03@caas.cn (R.Z.); 17899319024@163.com (L.Z.); anxuejiao@caas.cn (X.A.); chuneniu@163.com (C.N.); 3 Sheep Breeding Engineering Technology Research Center of Chinese Academy of Agricultural Sciences, Lanzhou 730050, China; 4 Institute of Agricultural Sciences of Qingyang, Qingyang 745000, China; 236xuzhenfei@163.com (Z.X.); gengzhiguang2@163.com (Z.G.); shihaina0716@163.com (H.S.); jinxia425@tom.com (J.Z.)

**Keywords:** sheep, crossbreeding, meat quality, nutritional composition, amino acids, fatty acids, volatile flavor compounds

## Abstract

With the increasing consumer demand for high-quality lamb, crossbreeding has become a key technology for improving the production performance and meat quality of sheep. To evaluate the meat quality advantages and characteristics of Suffolk (SFK) and Hu sheep (HH) and their F_1_ crossbreds (SH), thirty-six 3-month-old male lambs of SFK (*n* = 12), HH (*n* = 12), and SH (*n* = 12) were selected and raised in individual pens under the same nutritional and management conditions. After standardized feeding until 6 months of age, the *Longissimus dorsi* muscle was collected to determine meat quality traits, amino acid and fatty acid profiles, and volatile flavor compounds. The results indicated that the L*, a* and b* values of the SH group were significantly lower than those of the parental breeds (*p* < 0.05), with tenderness being intermediate between the two parent breeds. Notably, drip loss and cooking loss were significantly lower in the SH group (*p* < 0.05), indicating superior water-holding capacity. In terms of amino acid profiles, the contents of non-essential amino acids (NEAAs) and sweet-tasting amino acids in the SH group were significantly higher than those of the parent breeds (*p* < 0.05), with the overall profile meeting the FAO/WHO ideal protein pattern. Analysis of fatty acid profiles revealed that the SH group had significantly lower total saturated fatty acids (SFAs) (*p* < 0.05) and significantly higher levels of functional fatty acids (such as CLA), resulting in a significantly higher UFAs (unsaturated fatty acids)/SFAs (saturated fatty acids) ratio (*p* < 0.05) and superior nutritional value of fat. Furthermore, 32 volatile flavor compounds were detected in the SH group; among them, key aroma-active compounds such as isoamyl formate, 3-methyl-1-butanol, and acetoin were significantly higher than in the parental breeds (*p* < 0.05), contributing to a unique flavor profile. Consequently, this study systematically reveals the advantages of Suffolk × Hu F_1_ crossbreds in terms of meat quality, nutritional value, and flavor characteristics, providing fundamental data for the optimization of crossbreeding systems, breeding selection, and the quality improvement of sheep meat products.

## Simple Summary

Improving the eating quality and nutritional value of lamb is a priority for the meat industry to meet consumer demands. This study investigated the meat characteristics of offspring produced by crossing two distinct breeds: the Suffolk sheep (known for good muscle growth) and the Hu sheep (recognized for high reproductive rates). We compared the meat quality, nutrient composition, and flavor profiles of these crossbred sheep against their parent breeds. The results showed that the crossbred sheep produced meat with excellent tenderness and water-holding capacity, making it juicy and pleasant to eat. Nutritionally, the meat was rich in healthy fats and specific amino acids that provide a sweet taste. Furthermore, the analysis of aroma compounds revealed that the crossbred meat had a unique flavor profile that reduced the strong, sometimes unpleasant, odor often associated with lamb while boosting pleasant roasted and fruity aromas. In conclusion, crossing Suffolk and Hu sheep successfully combines the beneficial traits of both parent breeds, resulting in superior meat quality. This finding is valuable as it provides a scientific basis for breeding better sheep.

## 1. Introduction

With the improvement in living standards and increased health awareness, consumer demand for green, safe, nutritionally balanced, and uniquely flavored high-quality food is growing [1]. Lamb meat characterized by its high protein and low fat content, as well as being rich in unsaturated fatty acids and essential amino acids, has become a preferred choice for more consumers [2,3]. Although China is a major producer and consumer of lamb meat with abundant indigenous sheep resources, most of which possess inherent advantages such as strong adaptability, good stress resistance, and unique meat flavor [4], some also suffer from limitations, including low meat production performance, insufficient lean meat percentage, and suboptimal refined meat quality characteristics. Consequently, these breeds struggle to fully match the current market demand for high-quality lamb meat, making the cultivation of high-quality meat sheep and the optimization of meat quality regulation technologies urgently needed [5].

Crossbreeding is a key technology for improving livestock production performance and optimizing meat quality by fully utilizing the complementary effects between parent breeds and allowing offspring to aggregate excellent parental traits [6]. Xiao et al. [7] found that crossing Dorper and Australian White rams with Small-tailed Han ewes could improve the meat quality and flavor of the offspring, while Zhang et al. [8] observed that crossing Poll Dorset with Hu sheep enhanced the growth performance of the crossbred offspring. Overall, crossbreeding effectively improves the growth rate and meat production performance of the offspring.

Hu sheep, as an excellent indigenous meat breed in China, is characterized by tender meat and high prolificacy, making it an ideal maternal parent for crossbreeding [9,10]; however, it has disadvantages such as low production performance [11]. Suffolk sheep, an introduced superior meat breed, possesses the advantages of fast growth and high meat yield, making it a high-quality paternal parent for improving indigenous breeds [12]. Our previous studies have shown that the growth rate and meat production traits of Suffolk × Hu F_1_ crossbreds (SH) were superior to Hu sheep. However, the systematic differences in key quality indicators between the crossbred offspring and their parent breeds remain unclear [13]. Therefore, given the potential phenotypic and genetic variations in muscle growth, nutrient metabolism and flavor formation among different sheep breeds, we hypothesized that the *Longissimus dorsi* muscles of SH, Hu, and Suffolk sheep would exhibit significant discrepancies in multiple meat-quality-related indicators, and this study systematically compared the differences in meat quality traits, nutritional value, amino acid composition, fatty acid profiles, and volatile flavor compounds of the *Longissimus dorsi* among these three groups of sheep. These findings provide foundational data support for high-quality meat sheep breeding and the optimization of crossbreeding systems.

## 2. Materials and Methods

### 2.1. Experimental Animals and Sample Collection

This study was conducted at Gansu Qinghuan Meat Sheep Breeding Co., Ltd. (Huanxian, China). Thirty-six 3-month-old male lambs with similar birth dates were selected for the trial, including Suffolk (SFK, *n* = 12) and Hu sheep (HH, *n* = 12) and their F_1_ crossbreds (SH, *n* = 12). During the 95-day experimental period (including a 15-day adaptation period), the lambs were housed in individual pens under identical nutritional and management conditions. All animals were fed a total mixed ration (TMR) in pellet form and had ad libitum access to feed and water. Diets were formulated to meet feeding goals, and nutrient levels (on a dry matter basis) were calculated based on the Tables of Feed Composition and Nutritive Value in China [14]. The ingredients and chemical composition of the diets are listed in Table 1. At the end of the feeding trial, six lambs with body weights close to the group average were selected from each group and humanely slaughtered, and post hoc power analysis was conducted using the GPower 3.1 software (Heinrich Heine University Düsseldorf, Düsseldorf, Germany) with the parameters set as follows: effect size f = 0.9, α = 0.05, and number of groups = 3. The statistical power reached 0.87, which was higher than the threshold of 0.8, verifying that the sample size of 6 individuals per group was sufficient to ensure adequate statistical power. All animals were fasted for 12 h prior to slaughter and had free access to water. The experimental sheep were transported to the designated slaughterhouse over a distance of 500 m. During transportation, the environment was kept quiet to minimize stress. Upon arrival, the sheep were allowed to rest in a quiet environment for 2 h with ad libitum access to water. The animals were subjected to electrical stunning (220 V, 50 Hz, stunning duration 10 s) and then immediately slaughtered and exsanguinated following standard Islamic slaughter procedures. Within 45 min post-mortem, 400 g of *Longissimus dorsi* muscle sample was collected between the 12th and 13th ribs from each animal, with the sampling site completely consistent across all experimental animals. Of the total sample, 300 g was used for the immediate determination of meat quality traits (including pH, meat color, tenderness, etc.), while the remaining 100 g was stored at −80 °C for subsequent analysis of nutritional composition, amino acid profile, fatty acid profile, and volatile flavor compounds. The experimental design, procedures, and methods have been described previously [13] and were approved by the Institutional Animal Care and Use Committee of the Lanzhou Institute of Husbandry and Pharmaceutical Sciences, Chinese Academy of Agricultural Sciences (permit no. 2022-018), in compliance with Chinese standards for the care and use of research animals.

### 2.2. Measurement Indices and Methods

#### 2.2.1. Determination of Meat Quality

pH Value: The pH values of the *Longissimus dorsi* muscle at 45 min and 24 h post-mortem (denoted as pH_45min_ and pH_24h_) were determined using a portable pH meter (Testo 205, Testo AG, Lenzkirch, Baden-Württemberg, Germany) fitted with a penetration electrode. The electrode was inserted into the muscle at a depth of 1.5 cm. For each sample, triplicate measurements were performed at different positions, and the mean value was taken as the final result. Prior to each measurement, the pH meter was calibrated with standard buffer solutions of pH 4.01 and pH 7.00.

Tenderness: Tenderness was evaluated by measuring shear force. Muscle samples (*Longissimus dorsi*, trimmed into 5 cm × 3 cm × 3 cm cuboids) were wrapped in polyethylene bags and placed in a constant-temperature water bath at 80 °C. When the core temperature reached 70 °C monitored by a portable thermocouple thermometer, the samples were removed and cooled to room temperature under running tap water. Six cylindrical strips (1 cm × 1 cm × 3 cm) were cut parallel to the muscle fibers. Shear force was then measured using a muscle tenderness meter (Model RH-N50, Guangzhou Runhu Instruments Co., Ltd., Guangzhou, China), with each strip sheared perpendicular to the muscle fiber direction at a crosshead speed of 200 mm/min. Six replicates were recorded for each sample, and the mean value was calculated.

Cooked Meat Yield: Visible fascia and fat were removed from the *Longissimus dorsi* samples, and the initial weight was accurately recorded as *W*1. The samples were placed in a cooking pot with water preheated to 90 °C and cooked at this constant temperature for 25 min. After cooking, the samples were removed, cooled naturally to 25 °C, and blotted dry with filter paper. The final weight was recorded as *W*2. The cooked meat yield was calculated: cooked meat yield (%) = (*W*2/*W*1) × 100%.

Water Loss Rate: Muscle samples were trimmed into a cube (1 cm × 1 cm × 1 cm), and the initial weight was recorded as *W*3. The sample was placed between two layers of qualitative filter paper, with 18 layers of absorbent filter paper placed on the top and bottom, respectively. The assembly was placed in a pressure meter, and a pressure of 35 kg was applied for 5 min. The sample was then removed, and the final weight was recorded as *W*4. The water loss rate was calculated: water loss rate (%) = [(*W*3 − *W*4)/*W*3] × 100%.

Cooking Loss: Muscle samples were trimmed of fascia and fat, and the initial weight was recorded as *W*5. The samples were sealed in cooking bags and heated in a constant-temperature water bath (80 °C). When the core temperature reached 70 °C, it was maintained for 10 min. The heating was stopped, and the samples were cooled naturally to 25 °C. The samples were removed and blotted dry with filter paper, and the cooked weight was recorded as *W*6 (0.01 g precision). The cooking loss was calculated: cooking loss (%) = [(*W*5 − *W*6)/*W5*] × 100%.

Drip Loss: A muscle sample (50 g) was accurately weighed (*W*7) and suspended inside a plastic bottle using an S-hook, ensuring that the sample did not touch the bottle walls. The bottle was sealed and stored at 4 °C for 24 h. The sample was then removed and re-weighed (*W*8). The drip loss was calculated: drip loss (%) = [(*W*7 − *W*8)/*W*7] × 100%.

Meat Color: The lightness (L*), redness (a*), and yellowness (b*) of each sample were measured using a colorimeter (model WR-18, China Shenzhen Weifu Photoelectric Technology Co., Ltd., Shenzhen, China) according to standard operating procedures. Prior to measurement, fresh muscle samples were cut into 2 cm thick slices and aged for 30 min at 4 °C in the dark to allow for full oxygenation of myoglobin. For each sample, quintuplicate measurements were performed at different fat- and connective-tissue-free areas, and the mean value was taken as the final result.

#### 2.2.2. Determination of Nutritional Value

The contents of moisture, ash, crude protein, and crude fat in the samples were determined according to the National Food Safety Standards of China: (GB 5009.3-2016), (GB 5009.4-2016), (GB 5009.124-2016), (GB 5009.168-2016) [15,16,17,18].

The moisture content of lamb muscle samples was determined according to GB 5009.3-2016. Approximately 5.0 g of minced muscle sample (free of visible fat and connective tissue) was weighed into a pre-dried aluminum dish with sand and dried to a constant weight in a forced-air oven (Model DHG-9070A, Shanghai Jinghong Experimental Equipment Co., Ltd., Shanghai, China) at 103 ± 2 °C. The moisture content (%) was calculated as the weight loss divided by the initial sample weight.

The ash content was determined according to GB 5009.4-2016. Approximately 2–3 g of minced muscle sample was weighed into a pre-ignited and weighed crucible. The sample was carbonized on an electric hot plate (Model DB-3, Changzhou Guohua Electric Appliance Co., Ltd., Changzhou, China) and then incinerated in a muffle furnace (Model SX2-5-12A, Shanghai Yiheng Scientific Instrument Co., Ltd., Shanghai, China) at 550 ± 25 °C for at least 4 h until a constant weight was obtained. The ash content (%) was calculated as the ash mass divided by the initial sample mass.

#### 2.2.3. Determination of Amino Acids

The composition and content of amino acids were determined according to the National Food Safety Standard (GB 5009.124-2016) [17]. A muscle sample (50 mg) was accurately weighed into a hydrolysis tube, and 15 mL of hydrochloric acid solution (6 mol/L) was added and mixed. Nitrogen gas was slowly flushed into the hydrolysis tube for 2 min, after which the tube was quickly sealed. The sample was hydrolyzed in a constant-temperature oven (Model DHG-9140A, Shanghai Yiheng Scientific Instrument Co., Ltd., Shanghai, China) at 110 °C for 24 h. After cooling, the hydrolysate was filtered through quantitative filter paper into a 25 mL volumetric flask and diluted to volume with distilled water. An aliquot of 0.5 mL from the volumetric flask was transferred to a centrifuge tube and concentrated to near-dryness at 60 °C using a nitrogen evaporator (JL-DY12-N2Y, Hefei Jingli Instrument Equipment Co., Ltd., Hefei, China). To remove residual acid, 200 µL of ultrapure water was added, and the sample was concentrated to near-dryness again; this step was repeated twice. The residue was dissolved in 2.5 mL of hydrochloric acid solution (0.02 mol/L) and ultrasonicated for 5 min. After filtration through a 0.22 µm membrane filter, 1 mL of the filtrate was analyzed using an automatic amino acid analyzer (Biochrom 30+, Biochrom Ltd., Cambridge, UK).

Chromatographic Conditions: The analysis was performed using a PEEK/Na-type cation exchange column. The column temperature program ranged from 45 °C to 98 °C, and the reaction coil temperature was set at 135 °C. The buffer flow rate was 45 mL/h. The final volume for calculation was set to 100 mL, and the standard solution concentration was 0.25 µmol/mL.

#### 2.2.4. Determination of Fatty Acids

The composition and content of fatty acids were determined using the internal standard method, referencing the National Food Safety Standard (GB 5009.168-2016) [18]. Glycerol triundecanoate (C11:0) (Sigma-Aldrich, St. Louis, MO, USA) was used as the internal standard, and qualitative identification of target fatty acids was performed by comparing the retention time of chromatographic peaks in the samples with those of 37-component fatty acid methyl ester (FAME) mixed reference standards (Sigma-Aldrich, St. Louis, MO, USA).

Briefly, 10 g of muscle sample was placed in a moisture dish and dried in an oven (Model DHG-9140A, Shanghai Yiheng Scientific Instrument Co., Ltd., Shanghai, China) at 103 °C for 1 h. After cooling to room temperature in a desiccator (Shuniu Instrument Co., Ltd., Shanghai, China), the dried sample was ground into homogeneous powder. A 0.5 g portion of the meat powder was accurately weighed into a 10 mL glass centrifuge tube with a stopper, and 100 μL of 10 mg/mL internal standard (glycerol triundecanoate) solution was precisely added prior to extraction to ensure accurate quantification. Subsequently, 2 mL of a benzene–petroleum ether mixture (1:1, *v*/*v*, chromatographically pure) was added. The tube was tightly sealed and subjected to oscillation extraction for 24 h at room temperature in the dark to prevent lipid oxidation, which is a modified solvent extraction method for total lipid extraction from muscle tissue.

Subsequently, 2 mL of 0.4 mol/L potassium hydroxide–methanol solution (Aladdin Biochemical Technology Co., Ltd., Shanghai, China) was added to the tube for fatty acid methylation. The mixture was vortexed thoroughly for 3 min and then allowed to stand for 30 min at 25 °C to complete the base-catalyzed transesterification of total lipids into FAMEs. Ultrapure water was added up to the top of the tube, and the mixture was left to stand for 30 min to facilitate complete phase separation. Anhydrous sodium sulfate (Aladdin Biochemical Technology Co., Ltd., Shanghai, China) was added to the upper organic phase to remove residual moisture, and the supernatant was filtered through a 0.22 μm organic-phase filter membrane prior to instrumental analysis. An aliquot of 100 μL of the filtered supernatant was collected, diluted to 1 mL with chromatographically pure n-hexane (Aladdin Biochemical Technology Co., Ltd., Shanghai, China), and analyzed using a gas chromatography system equipped with a flame ionization detector (Agilent Technologies 7890A, Santa Clara, CA, USA).

The gas chromatography conditions were as follows: An Agilent HP-88 capillary column (100 m × 0.25 mm inner diameter × 0.20 μm film thickness, specifically optimized for FAME separation) (Agilent Technologies, Santa Clara, CA, USA) was used. The column oven temperature program was initiated at 100 °C and held for 13 min; increased to 180 °C at a rate of 10 °C/min and held for 20 min; increased to 200 °C at a rate of 1 °C/min and held for 20 min; and finally increased to 230 °C at a rate of 4 °C/min and held for 10.5 min. The injector temperature was set at 250 °C, and the flame ionization detector (FID) temperature was maintained at 280 °C. High-purity nitrogen (N2, ≥99.999%) was used as the carrier gas at a constant flow rate of 1.0 mL/min, with a split ratio of 100:1. Samples were injected using an automatic split sampler with an injection volume of 1 μL. The quantitative calculation of each fatty acid was performed using the internal standard method based on the peak area ratio of the target FAME to the internal standard.

#### 2.2.5. Determination of Volatile Flavor Compounds

The composition and content of volatile flavor compounds were determined using a FlavourSpec^®^ flavor analyzer (G.A.S., Dortmund, North Rhine-Westphalia, Germany). A 2 g portion of minced and homogenized muscle sample (passed through a 40-mesh sieve to ensure uniformity) was accurately weighed into a 20 mL headspace vial sealed with a polytetrafluoroethylene (PTFE)/silicone septum and subjected to static headspace equilibration by incubation at 60 °C for 15 min (equilibration time) with continuous agitation at a speed of 500 rpm. Subsequently, 500 µL of the headspace gas was automatically injected in splitless mode using a heated gas-tight syringe. The syringe temperature was maintained at 80 °C, and high-purity nitrogen (N_2_, ≥99.999%) was used as the carrier gas.

GC-IMS Conditions: Chromatographic separation was performed using an FS-SE-54-CB-1 capillary column (15 m × 0.53 mm inner diameter × 1.0 μm film thickness, with a stationary phase of 5% phenyl–95% methyl polysiloxane) (Agilent Technologies, Santa Clara, CA, USA). The column temperature was set at a constant 60 °C, and the total analysis time was 20 min. The IMS drift tube temperature was maintained at 45 °C, with a tritium (^3^H) ionization source used for compound ionization. The carrier gas flow rate was programmed as follows: initially held at 2 mL/min for 2 min, linearly increased to 100 mL/min over the next 8 min (2–10 min), and maintained at 100 mL/min for the final 10 min (10–20 min). High-purity nitrogen (N_2_, ≥99.999%) was used as the drift gas with a constant flow rate of 150 mL/min.

Data Analysis: Quantitative analysis of volatile flavor compounds was performed using the built-in VOCal software (Version 1.0, G.A.S., Dortmund, North Rhine-Westphalia, Germany), and relative quantitative analysis was carried out using the peak area normalization method, with the relative content of each target compound expressed as the percentage of its peak area in the total peak area of all identified volatile compounds. Qualitative analysis was conducted by matching the retention index (RI) and drift time of detected substances against the built-in NIST gas chromatography retention index database and IMS drift time database of the instrument. The RI was pre-calibrated using a mixture of n-alkane standards (C6–C20) (Sigma-Aldrich, St. Louis, MO, USA), with a retention index matching tolerance of ≤±50 index units and a relative drift time matching deviation of ≤±2% set as the criteria for positive compound identification. The Reporter plug-in was used to compare spectral differences between samples, the Gallery Plot plug-in was used to generate fingerprint comparisons of volatile compounds, and the Dynamic PCA plug-in was used for unsupervised Principal Component Analysis (PCA) to visualize sample clustering differences.

### 2.3. Statistical Analysis

Data were preliminarily processed using Microsoft Excel 2019 (Microsoft Corporation, Redmond, WA, USA). Prior to formal statistical testing, all experimental data were subjected to normality testing via the Shapiro–Wilk method and homogeneity of variance testing via Levene’s method. Data conforming to the assumptions of normal distribution and homogeneity of variance were subjected to subsequent parametric statistical analysis, while data failing to meet the test hypotheses were analyzed using the non-parametric Kruskal–Wallis test. Statistical analysis was performed using the SPSS 26.0 software (IBM Corp., Armonk, NY, USA) and R 4.3.0 software (R Core Team, Vienna, Austria). For data meeting parametric assumptions, one-way analysis of variance (ANOVA) was adopted to evaluate differences among groups, followed by Duncan’s multiple range test for post hoc comparisons. All multiple comparison results were corrected for false discovery rate (FDR) using the Benjamini–Hochberg method to reduce the risk of type I error caused by multiple testing. All experimental results are presented as mean ± standard deviation (SD). Differences were considered statistically significant at *p* < 0.05.

## 3. Results

### 3.1. Comparison of Meat Quality Traits of the Longissimus dorsi for SH and Their Parental Breeds

The results of the meat quality traits for the Suffolk × Hu F_1_ crossbreds (SH) and their parent breeds are summarized in Table 2. There were no significant differences in the initial pH_45min_ and ultimate pH_24h_ among the SH, SFK, and HH groups (*p* > 0.05). The L* and a* values of the SH group were significantly lower than those of the SFK and HH groups (*p* < 0.05). The b* value of the SH group was significantly lower than that of the SFK group, which, in turn, was significantly lower than that of the HH group (*p* < 0.05). The tenderness of the SH group was significantly lower than that of the SFK group but significantly higher than that of the HH group (*p* < 0.05). For processing characteristics, no significant difference was observed in cooked meat yield among the three groups. However, the drip loss was significantly different (*p* < 0.05), with the SFK group exhibiting the highest value, followed by the HH group, and with the SH group exhibiting the lowest. Significant differences were also observed in water loss rate (*p* < 0.05), which was highest in the SH group, followed by the SFK group, and it was lowest in the HH group. Regarding cooking loss, the SH group was significantly lower than both the SFK and HH groups (*p* < 0.05).

### 3.2. Comparison of Nutritional Value of the Longissimus dorsi for SH and Their Parental Breeds

The results of the comparative analysis concerning the nutritional value of the *Longissimus dorsi* muscle for the SH, SFK, and HH groups are presented in Table 3. Regarding moisture content, significant differences were observed among the three groups (*p* < 0.05); the SH group exhibited the lowest moisture content, followed by the HH group, with the SFK group being the highest. No significant differences were found in the contents of ash, crude protein, or crude fat among the three groups (*p* > 0.05). However, a numerical trend was observed for crude protein content in the order of SH > SFK > HH (*p* = 0.06).

### 3.3. Comparison of Amino Acid Profiles of the Longissimus dorsi for SH and Their Parental Breeds

The comparative results of the amino acid profiles in the *Longissimus dorsi* muscle are presented in Table 4. A total of 17 amino acids were detected across the three groups, including 7 essential amino acids (EAAs) and 10 non-essential amino acids (NEAAs). Statistical analysis revealed no significant differences in the contents of total essential amino acids (EAAs) and total amino acids (TAAs) between SH group and its parental groups (*p* > 0.05). However, the content of NEAAs in the SH group was significantly higher than that in the SFK group and HH group, and the SFK group was significantly higher than the HH group (*p* < 0.05). The ratios of EAAs/TAAs and EAAs/NEAAs showed no significant differences among the three groups. Regarding specific EAA components, no significant differences were observed in the contents of any individual essential amino acids (such as leucine, lysine, valine, etc.) among the three groups (*p* > 0.05). Regarding specific NEAA components, no significant differences were found in the contents of glutamic acid, aspartic acid, arginine, proline, tyrosine, histidine, or cysteine (*p* > 0.05). However, significant differences were observed in serine, glycine, and alanine. Specifically, the contents of serine and glycine in the SH group were significantly higher than those in the SFK group and HH group (*p* < 0.05), while no significant difference was found between the two parental groups. For alanine, the SH group was significantly higher than the SFK group, which, in turn, was significantly higher than the HH group (*p* < 0.05). In terms of flavor-presenting amino acids, no significant differences were observed in the contents of bitter amino acids (BAAs) and umami amino acids (UAAs) among the three groups (*p* > 0.05). However, significant differences were found in sweet amino acids (SAAs). The content of SAAs in the SH group was significantly higher than in the SFK group, and that in the SFK group was significantly higher than in the HH group (*p* < 0.05).

### 3.4. Comparison of Fatty Acid Profiles of the Longissimus dorsi for SH and Their Parental Breeds

The comparison of the fatty acid profiles of the *Longissimus dorsi* muscle is presented in Table 5. A total of 26 fatty acids were detected in the three groups, including 10 saturated fatty acids (SFAs), 6 monounsaturated fatty acids (MUFAs), and 10 polyunsaturated fatty acids (PUFAs).

In terms of saturated fatty acids, the content of caprylic acid in the SH group was significantly lower than that in the SFK and HH groups (*p* < 0.05). The content of myristic acid was significantly higher in the HH group compared to the SFK and SH groups (*p* < 0.05). The content of arachidic acid was significantly higher in the HH group than in the SFK group, which, in turn, was significantly higher than in the SH group (*p* < 0.05). Although the contents of capric acid, lauric acid, tridecanoic acid, pentadecanoic acid, and palmitic acid in the SH group were lower than those in the SFK and HH groups and stearic acid was lower than in the HH group, these differences did not reach statistical significance (*p* > 0.05). No significant differences were observed in the content of margaric acid among the three groups.

Regarding monounsaturated fatty acids, the content of myristoleic acid in the SH group was significantly lower than that in the SFK and HH groups (*p* < 0.05). The content of gadoleic acid in the SH group was significantly lower than in the SFK group, which was significantly lower than in the HH group (*p* < 0.05). Conversely, the content of erucic acid in the SH group was significantly higher than in the SFK group, which was significantly higher than in the HH group (*p* < 0.05). The contents of palmitoleic acid, nervonic acid, and oleic acid in the SH group were lower than those in the parental groups, but the differences were not statistically significant (*p* > 0.05).

For polyunsaturated fatty acids, the content of conjugated linoleic acid (CLA) in the SH group was significantly higher than in the HH group, which was significantly higher than in the SFK group (*p* < 0.05). The content of dihomo-γ-linolenic acid (DGLA) in the SH group was significantly lower than in the HH group, which was significantly lower than in the SFK group (*p* < 0.05). The content of arachidonic acid (AA) in the SH group was significantly lower than that in both the SFK and HH groups (*p* < 0.05). The content of cis-13,16-docosadienoic acid in the SH group was significantly lower than in the SFK group, which was significantly lower than in the HH group (*p* < 0.05). The contents of linoleic acid (LA), eicosadienoic acid, eicosapentaenoic acid (EPA), and docosahexaenoic acid (DHA) in the SH group were lower than those in the SFK and HH groups, but the differences were not significant (*p* > 0.05). No significant differences were found in the contents of γ-linolenic acid (GLA) and α-linolenic acid (ALA) among the three groups.

The comparison of the total fatty acids and their proportions is presented in Table 6. The contents of total saturated fatty acids (SFAs), total polyunsaturated fatty acids (PUFAs), and total fatty acids (TFAs) in the SH group were significantly lower than those in the SFK and HH groups (*p* < 0.05). Conversely, the ratio of UFAs/SFAs in the SH group was significantly higher than that in both parental breeds (*p* < 0.05). Regarding specific fatty acid series, the content of n-3 PUFAs in the SH group was significantly lower than in the SFK and HH groups (*p* < 0.05). However, no significant differences were observed among the three groups in terms of total monounsaturated fatty acids (MUFAs), total unsaturated fatty acids (TUFAs), n-6 PUFAs, or the n-6/n-3 ratio (*p* > 0.05).

### 3.5. Comparison of Volatile Flavor Compound Contents in the Longissimus dorsi for SH and Their Parental Breeds

**Figure 1:**
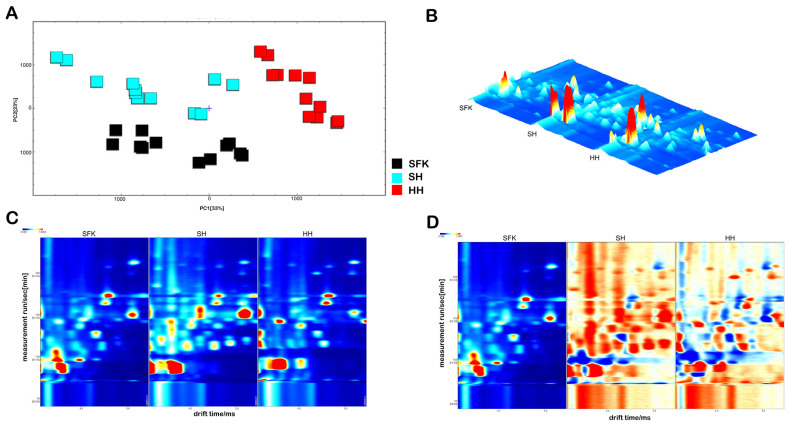
(**A**) PCA plot of volatile compounds; (**B**) GC-IMS 3D topographic plot; (**C**) GC-IMS 2D spectrum; (**D**) GC-IMS difference comparison plot.

Gas chromatography–ion mobility spectrometry (GC-IMS) combined with multivariate statistical analysis was employed to investigate the breed-specific differences in volatile flavor compounds of the *Longissimus dorsi* muscle among the SH, SFK, and HH groups. As illustrated in Figure 1A, Principal Component Analysis (PCA) revealed that PC1 (33%) and PC2 (20%) accounted for a cumulative variance of 53%. The samples within each group clustered tightly, while the clustering regions between groups showed no overlap (with the SFK group concentrated in the lower left, the SH group in the middle, and the HH group in the upper right), indicating that PCA could effectively distinguish the volatile flavor profiles of the three groups. The GC-IMS 3D topographic plot (Figure 1B) and the original 2D spectrum (Figure 1C) showed highly overlapping peak distributions among the three groups, suggesting similarity in the types of volatile compounds present. However, significant differences in the signal intensities of certain ion peaks were observed, indicating breed-specific variations in the contents of volatile flavor compounds between the crossbred sheep and their parent breeds. The difference comparison plot (Figure 1D), through contrast enhancement, visually highlights the differences in signal intensity among the breeds; red/orange regions represent a higher content of the corresponding substance in a specific breed, while blue regions indicate a lower content, clearly distinguishing the characteristic signal regions of the SFK, SH, and HH groups.

**Figure 2:**
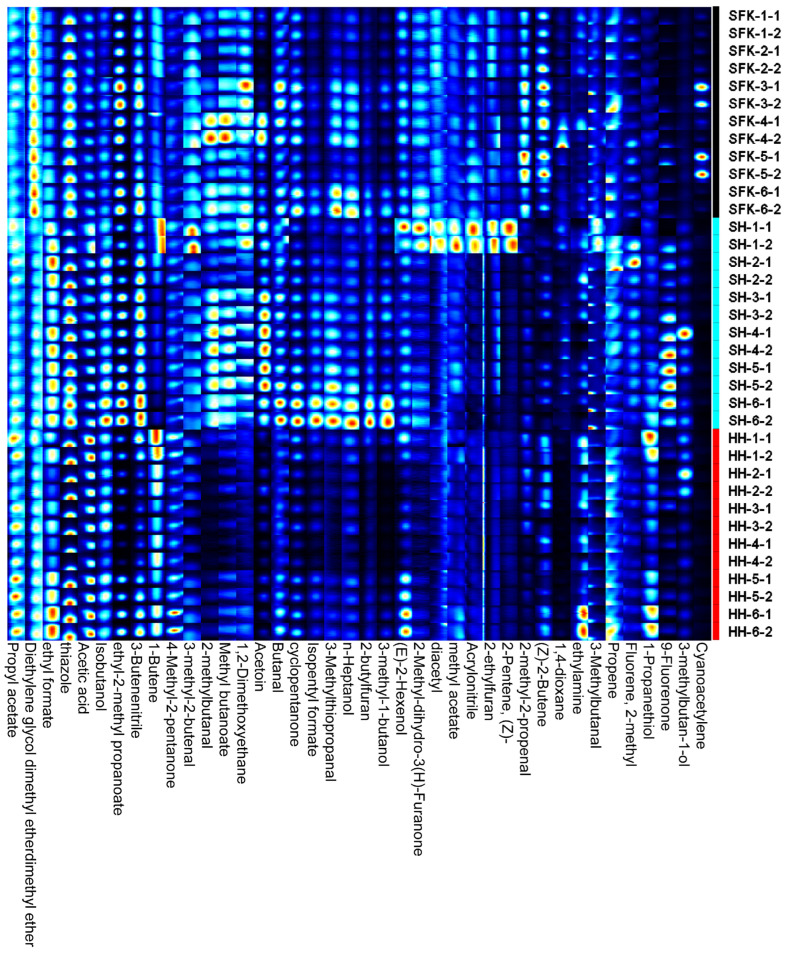
Fingerprints of volatile compounds.

To comprehensively and visually compare the differences in volatile flavor compounds of the *Longissimus dorsi* muscle among the SH, SFK, and HH groups, the Gallery Plot plug-in was used to select all signal peaks for analysis from the GC-IMS 2D spectrum, automatically generating a volatile compound fingerprint (Figure 2). This plot simplifies multidimensional data into a visualized matrix. The signal patterns of intra-group replicates were highly similar, whereas inter-group samples exhibited characteristic signal differences. Specifically, the contents of 4-methyl-2-pentanone, ethyl 2-methylpropanoate, thiazole, ethyl formate, and propyl acetate were relatively similar across the three groups. In contrast, isoamyl formate, 3-methyl-1-butanol, and acetoin were identified as characteristic flavor compounds in the SH group.

The qualitative analysis of volatile flavor compounds in all samples is presented in Table 7. A total of 32 volatile compounds were definitively identified. Regarding esters, the contents of isoamyl formate and ethyl formate in the SH group were significantly higher than those in the SFK and HH groups (*p* < 0.05). Conversely, the contents of ethyl 2-methylpropanoate and methyl butanoate were significantly higher in the SFK group compared to the SH and HH groups (*p* < 0.05). Additionally, propyl acetate was found to be highest in the HH group, followed by the SH group, and it was lowest in the SFK group (*p* < 0.05). In the category of alcohols, the levels of isobutanol and 3-methyl-1-butanol in the SH group were significantly higher than those in both the SFK and HH groups (*p* < 0.05). No significant differences were observed for n-heptanol or (E)-2-hexenol among the three groups. As for ketones, the contents of acetoin and 9-fluorenone in the SH group were significantly higher than in the SFK and HH groups (*p* < 0.05). However, the content of 4-methyl-2-pentanone was significantly higher in the HH group (*p* < 0.05). Furthermore, the contents of cyclopentanone and 2-methyl-dihydro-3(H)-furanone were significantly highest in the SFK group (*p* < 0.05). In terms of acids, the content of acetic acid was significantly higher in the HH group than in the SH group, which, in turn, was significantly higher than in the SFK group (*p* < 0.05). Regarding aldehydes, the SH group exhibited significantly higher levels of 3-methylthiopropanal and 2-methylbutanal compared to the SFK and HH groups (*p* < 0.05). In contrast, the contents of 3-methyl-2-butenal and 2-methyl-2-propenal were significantly higher in the SFK group (*p* < 0.05). For thiazoles, unlike other non-significant compounds, the content of thiazole was significantly highest in the SFK group, followed by the SH group, and it was lowest in the HH group (*p* < 0.05). For hydrocarbons and other compounds, the level of 2-methylfluorene in the SH group was significantly higher than in both the SFK and HH groups, while the content of (Z)-2-butene was significantly higher in the SFK group (*p* < 0.05). Additionally, 2-butylfuran was significantly highest in the SH group, whereas 1-propanethiol was significantly highest in the HH group (*p* < 0.05). No significant differences were observed for (Z)-2-pentene, propene, 1-butene, or 2-ethylfuran among the three groups.

## 4. Discussion

### 4.1. Meat Quality Traits of the Longissimus dorsi for SH and Their Parental Breeds

The pH value is a critical indicator reflecting the post-mortem freshness of meat, and its stability directly affects processing characteristics and storage potential [19]. High-quality lamb meat typically exhibits a pH_45min_ between 6.0 and 6.8 and a pH_24h_ between 5.4 and 5.8. Within this range, the post-mortem glycolysis process is stable, ensuring a strong capacity for maintaining freshness [20]. In this study, no significant differences were observed in pH_45min_ and pH_24h_ among the SH, SFK and HH groups, and all values fell within the optimal range for high-quality lamb meat. This finding suggests that the SH group inherited the excellent genetic trait of stable post-mortem glycolysis from their parental breeds, possessing a freshness maintenance capacity consistent with that of the parental breeds, which lays a good foundation for storage and processing. This result aligns with the findings of Quan et al. [21] regarding the pH stability of offspring from Suffolk and Small-tailed Han sheep crosses, confirming the genetic conservation of core freshness indicators in crossbred sheep. This characteristic is closely related to the stable expression of genes associated with post-mortem muscle energy metabolism [22].

Meat color is the primary sensory indicator for consumers evaluating lamb meat quality. It is mainly determined by the content and oxidation state of myoglobin, with L* (lightness), a* (redness), and b* (yellowness) serving as core quantitative parameters that directly influence purchase intention [23]. In this study, compared to the bright red color of the SFK group and the darker color of the HH group, the intermediate color characteristic of the SH group aligns better with the preference of certain consumers for high-quality dark lamb meat, significantly enhancing its market acceptance and constituting a key commercial advantage. This difference is likely related to the expression of genes involved in myoglobin synthesis during hybridization, resulting in a myoglobin content in the SH group that is intermediate between the parent breeds but skewed towards a lower level [24], further validating the precise improvement effect of crossbreeding on meat sensory traits.

Tenderness is a core indicator determining the eating quality of lamb meat and directly affecting consumer experience. It is primarily associated with muscle fiber diameter, intramuscular collagen content, and the degree of cross-linking; a lower shear force value indicates more tender meat [25,26]. In this study, the tenderness of the SH group was significantly lower than that of the SFK group but higher than that of the HH group, exhibiting a typical intermediate inheritance pattern. The crossbred offspring balanced the muscle fiber characteristics of both parent breeds through genetic recombination, avoiding the disadvantage of poor tenderness in the SFK group while inheriting the advantage of fine fibers from the HH group, making the tenderness intermediate between the two parent breeds. This differentiated tenderness satisfies the core consumer demand for a tender texture while distinguishing itself from the overly soft texture of the HH group, possessing broader market adaptability. This fully reflects the directional optimization effect of crossbreeding on tenderness, consistent with the conclusion that “hybrid progeny exhibited heterosis for tenderness, surpassing the average value of the two parental breeds.” [27].

Processing characteristics such as drip loss, the water loss rate, and cooking loss are directly linked to nutrient loss and yield efficiency during lamb meat processing. Drip loss and the water loss rate reflect the water-holding capacity (WHC) of the muscle; a stronger WHC indicates better juiciness, higher processing yield, and superior eating quality [28,29]. In this study, the SH group exhibited significantly lower drip loss and cooking loss compared to their parental breeds, indicating that the WHC of the SH group is significantly superior to that of their parental breeds, representing a key heterosis advantage. Muscle WHC is closely related to its sarcoplasmic protein content, muscle tissue structure, and pH value [30]. It is speculated that the SH group possesses differentiated advantages in sarcoplasmic protein synthesis or muscle cell structure, thereby enhancing its water-holding performance. Notably, the contradiction between the highest water loss rate in the SH group and its superior WHC (low drip/cooking loss) may be attributed to the more severe mechanical conditions (pressure and time) used in the water loss rate determination, which might cause more significant damage to the muscle tissue structure. The underlying regulatory mechanism warrants further investigation combining histological observation and molecular biology techniques, pointing toward a direction for future research.

### 4.2. Nutritional Value of the Longissimus dorsi for SH and Their Parental Breeds

Moisture, crude protein, crude fat, and ash are fundamental indicators for evaluating the nutritional value of meat, and their proportions directly determine the nutrient density and edible value of the product [31]. In this study, moisture content followed the trend of SFK group > HH group > SH group, with the SH group being significantly lower than both parent breeds. Since moisture content is negatively correlated with dry matter content, this differentiated characteristic implies that the dry matter (protein and fat) in the SH group is relatively more concentrated, resulting in higher nutrient density. Further analysis reveals that the SH group exhibited a crude protein content numerically higher than both parent breeds and a crude fat content intermediate between them. This characteristic inherits the parental advantages of high protein from the SFK group and high fat from the HH group while achieving an optimized balance of “high protein and low fat.” This precisely fits modern consumer demand for healthy meat products [32], highlighting its nutritional advantage. These differentiated nutritional characteristics verify the effectiveness of the Suffolk × Hu sheep crossbreeding combination in nutritional optimization, providing practical evidence for directional breeding of high-quality lamb meat sheep.

Ash content is primarily composed of minerals, directly reflecting the mineral supply capacity of the meat [33]. The lack of a significant difference in ash content among the three groups indicates that the mineral supply capacity of the SH group is consistent with their parental breeds, satisfying basic human mineral requirements. Combined with the moisture, crude protein and crude fat analysis, the SH group has achieved a concentration of nutritional value through the differentiated regulation of moisture content while inheriting the excellent nutritional traits of their parental breeds, further confirming the heterosis of the Suffolk × Hu combination.

### 4.3. Amino Acid Composition of the Longissimus dorsi for SH and Their Parental Breeds

Amino acids are the basic units of protein, and their composition and content in muscle are important indicators for evaluating meat quality and protein nutritional value [34]. According to the FAO/WHO recommended pattern, high-quality protein is characterized by a ratio of essential amino acids to total amino acids (EAAs/TAAs) of approximately 40% and a ratio of essential amino acids to non-essential amino acids (EAAs/NEAAs) above 60% [35]. In this study, the EAA/TAA ratios for the SFK, SH, and HH groups were 40.08%, 38.93%, and 39.53%, respectively, and the EAA/NEAA ratios were 66.90%, 63.74%, and 65.38%, respectively. All values align well with the FAO/WHO recommended pattern, indicating that the amino acid composition of lamb meat from all three groups is excellent and qualifies as high-quality protein. Regarding specific differences, the core distinction lies in the content of non-essential amino acids (NEAAs). The SH group exhibited a significant “transgressive heterosis” in NEAA content, which was significantly higher than that of both parental breeds. Specifically, serine, glycine, and alanine in the SH group were significantly higher than in the SFK and HH groups. Although NEAAs are not essential for the human body, they participate in physiological processes such as metabolic regulation and immune function enhancement [36]. Moreover, these specific NEAAs are crucial flavor precursors; their differentiated increase positively influences both the flavor and nutritional value of the SH group. The lack of a significant difference in EAA content compared to the parent breeds suggests that the SH group, while maintaining the parental advantage of EAA supply, has formed a unique nutritional advantage through the specific accumulation of NEAAs. This characteristic is speculated to stem from the synergistic expression or heterosis of genes related to amino acid synthesis in the F_1_ generation [37]. Amino acids are a primary source of meat flavor substances. The types of amino acids in muscle are closely related to meat quality and flavor, with the content of flavor-presenting amino acids directly determining the taste profile [38]. Glutamic acid and aspartic acid are associated with umami (freshness), while threonine, serine, glycine, proline, and lysine are associated with sweetness [39]. Higher contents of these amino acids contribute to a more delicious taste. Conversely, valine, isoleucine, and leucine are associated with bitterness [40]. In this study, no significant differences were found in umami or bitter amino acid contents between SH and their parental breeds. However, a key finding is that the content of sweet amino acids (SAAs) in the SH group was significantly higher than in both the SFK and HH groups. This is primarily driven by the significant enrichment of glycine, alanine, and serine, which are known to impart sweet and savory notes to meat. This result indicates that crossbreeding improvement has significantly enhanced the meat flavor profile of the offspring, specifically by improving the sweetness characteristics. This is consistent with the findings of Zhang et al. [41] regarding the advantage of flavor amino acids in crossbred sheep. Comprehensively considering amino acid nutrition and flavor, the SH group exhibits superior quality, distinct sweet flavor characteristics, and higher nutritional value, with an amino acid composition that better meets consumer demands for palatable meat. The differentiated advantage of the SH group in NEAA and sweet amino acid contents directly confirms the effectiveness of crossbreeding. The recombination of excellent genes from both parent breeds during hybridization likely activated superior genes related to the synthesis of sweet-tasting amino acids, thereby forming a differentiated flavor advantage [42]. This provides a key theoretical basis and technical support for flavor-oriented objectives in sheep crossbreeding.

### 4.4. Fatty Acid Composition of the Longissimus dorsi for SH and Their Parental Breeds

The composition and content of fatty acids in muscle directly affect lamb meat quality and nutritional value, serving as important precursors for meat flavor [43]. Fatty acids are categorized into saturated fatty acids, monounsaturated fatty acids, and polyunsaturated fatty acids. Since different types of fatty acids have significantly different effects on human health, their composition ratio is a core basis for evaluating the nutritional value of lamb meat [44]. In this study, the SH group shared the same types of fatty acids with their parental breeds but exhibited significant differences in specific component contents and totals. These differences, specifically the variations in SFAs, MUFAs, and PUFAs, as well as the specific accumulation of functional fatty acids, collectively constitute the fatty acid nutritional advantage of the SH group. Research indicates a strong positive correlation between SFA intake and the prevalence of cardiovascular diseases [45]; thus, reducing SFA content in lamb meat is particularly important. In this study, the SFA content in the SH group was significantly lower than that in the SFK and HH groups (except for margaric acid, which was consistent with the parent breeds). This result suggests that crossbreeding can effectively reduce the health-unfavorable SFA content, enhancing the health value of the meat. Stearic acid (C18:0) is a key component contributing to the specific odor (“muttony” smell) of sheep meat, and its content is positively correlated with this odor [46]. In this study, the stearic acid content in the SH group was numerically lower than in the SFK group, suggesting that crossbreeding might reduce the lamb meat odor and further improve flavor. Compared to SFAs, PUFAs play roles in regulating blood lipids, reducing blood viscosity, and enhancing immunity, while MUFAs contribute positively to preventing atherosclerosis and coronary heart disease [47]. The SH group exhibited a differentiated characteristic of “high functional fatty acids and low non-functional fatty acids.” Regarding MUFAs, the content of erucic acid (C22:1) in the SH group was significantly higher than in the parent breeds, while myristoleic acid and gadoleic acid were significantly lower. The differentiated increase in long-chain MUFAs further enhances its nutritional value [48]. Regarding PUFAs, the content of conjugated linoleic acid (CLA) in SH group was significantly higher than in the parent breeds. CLA is a recognized functional fatty acid with physiological functions such as antioxidant, anti-inflammatory, and anti-obesity effects [49]. Its differentiated advantage significantly elevates the nutritional value of the SH group. Oleic acid (C18:1n9c) was the most abundant MUFA in this study and is known for its lipid-lowering effects [50]. The oleic acid content in the SH group was intermediate between the SFK and HH groups, with no significant difference. Although the total fatty acid content and the content of various fatty acid classes in the SH group were generally lower than in the parent breeds, the ratio of UFAs/SFAs in the SH group was significantly higher (1.43). The UFA/SFA ratio is a core evaluation indicator for fat nutritional value; meat with a UFA/SFA ratio > 1.0 is generally considered more aligned with healthy dietary standards [51]. This differentiated characteristic, combined with the advantage of a low SFA content, fully reflects the heterosis in fatty acid nutrition of the SH group.

### 4.5. Volatile Flavor Compounds of the Longissimus dorsi for SH and Their Parental Breeds

The flavor of lamb meat and its products is a crucial factor influencing consumer purchasing power and satisfaction. It is composed of a variety of compounds produced through the interaction of low-molecular-weight water-soluble substances and lipid oxidation products via the Maillard reaction [52,53]. In this study, gas chromatography–ion mobility spectrometry (GC-IMS) combined with multivariate statistical analysis was used to clarify the differences in volatile flavor compounds between SH and their parental breeds. Principal Component Analysis (PCA) showed that the three groups clustered tightly without overlapping, indicating that the SH group possesses unique volatile flavor characteristics distinguishable from their parental breeds. The GC-IMS spectra confirmed that while the types of compounds were similar across groups, their contents differed significantly, laying the foundation for the formation of the unique flavor advantage of the SH group. A total of 32 volatile compounds were identified in the lamb meat samples from the three groups. Overall, the SH group exhibited significantly higher contents of various volatile substances compared to the SFK and HH groups. Specifically, the SH group showed significantly higher levels of esters (e.g., ethyl formate, isoamyl formate). These substances typically possess pleasant fruity and sweet aromas, which can modify the lamb meat odor and improve flavor acceptability. Additionally, ketones such as acetoin and 4-methyl-2-pentanone were significantly elevated (or specific to) the crossbred or parental groups. Notably, acetoin, a compound with typical creamy and buttery notes derived from the Maillard reaction or lipid oxidation, was significantly highest in the SH group, contributing to a richer fatty aroma [54,55]. Regarding alcohols, isobutanol was significantly higher in the SH group. Although some alcohols have high detection thresholds, they are produced by the decomposition of fatty acid secondary hydroperoxides and act synergistically with aldehydes generated from unsaturated fatty acid oxidation, playing a vital role in forming the overall flavor base. Aldehydes, due to their low olfactory thresholds, are the most important contributors to meat flavor [54]. The results showed that 2-methylbutanal and 3-methylthiopropanal in the SH group were significantly higher than in the other groups. 2-Methylbutanal, derived from the Strecker degradation of isoleucine, possesses typical nutty, malty, and roasted aromas [56], suggesting that muscle of the SH group may contain richer free amino acid precursors that underwent more intense Maillard reactions during heating. Meanwhile, the significantly higher content of methional in the SH group is critical, as this compound is key to producing “cooked meat” and “potato” flavors, forming the core skeleton of meat’s characteristic aroma [57]. Furthermore, the detection of thiazoles and pyrazines further confirmed that complex non-enzymatic browning reactions occurred in samples of the SH group during heating. These nitrogen- and sulfur-containing heterocyclic compounds provide roasted meat aromas and specific savory notes [58]. The SH group outperformed the SFK and HH groups in the profile of certain volatile flavor compounds. In particular, the significant enrichment of key flavor substances—such as branched-chain aldehydes and sulfur-containing compounds derived from Strecker degradation, as well as esters and ketones derived from lipid oxidation—collectively endowed the lamb meat of the SH group with fuller meat and fat aroma characteristics. The formation of this flavor advantage is speculated to be closely related to the breed-specific metabolic regulation characteristics, muscle fiber type composition, and intramuscular fat deposition ability of the SH group. These factors likely facilitated the accumulation of flavor precursors such as reducing sugars, free amino acids, and unsaturated fatty acids in the muscle tissue, providing a sufficient basis for the subsequent generation of flavor compounds.

## 5. Conclusions

Through crossbreeding, Suffolk × Hu F_1_ crossbreds inherited the excellent characteristics of both parent breeds and exhibited significant heterosis in meat quality, nutritional composition, amino acid profile, fatty acid spectrum, and volatile flavor compounds, making it an ideal crossbreeding combination for high-quality lamb meat sheep breeding. The results of this study provide important theoretical basis and data support for the optimization of lamb meat sheep crossbreeding systems.

## Figures and Tables

**Table 1:** Composition of total mixed ration and its nutritional levels on a dry matter basis.

Ingredients	Content	Nutrient Level	Content
Corn	40.00	Dry Matter (DM)	88.68
Soybean meal	6.00	Crude Protein (CP)	15.88
Wheat bran	4.50	Ether Extract (EE)	3.08
Cottonseed meal	4.60	Crude Ash (Ash)	7.24
Corn germ meal	5.30	Metabolic Energy (MJ/kg)	11.65
Malt root	5.00	Acid Detergent Fiber (ADF)	16.99
DDGS	6.00	Neutral Detergent Fiber (NDF)	31.50
Molasses	3.50	Calcium (Ca)	0.97
Straw meal pellet	20.00	Phosphorus (P)	0.49
Sodium bicarbonate	1.00		
Calcium carbonate	1.20		
200 slow-release urea	0.50		
Sodium chloride	0.60		
Calcium hydrogen phosphate	0.50		
Magnesium oxide	0.10		
Yeast culture	0.50		
1% premix	0.70		
Total	100.00		

Content of 1% premix: Cu: 1300 mg/kg, Fe: 6000 mg/kg, Mn: 4500 mg/kg, Zn: 8500 mg/kg, I: 170 mg/kg, Se: 45 mg/kg, Co: 65 mg/kg, Vit A 800 KIU/kg, Vit D3 350 KIU/kg, Vit E 7000 IU/kg.

**Table 2:** The results of meat quality indicators of the *Longissimus dorsi* for SH and their parental breeds (*n* = 6).

Item	SFK	SH	HH	*p*-Value
pH (pH_45min_)	6.42 ± 0.15	6.45 ± 0.09	6.61 ± 0.13	0.06
pH (pH_24h_)	5.45 ± 0.05	5.52 ± 0.02	5.45 ± 0.08	0.07
L*	42.39 ± 2.00 ^a^	33.19 ± 1.02 ^c^	41.57 ± 0.58 ^b^	0.01
a*	22.07 ± 0.67 ^a^	17.04 ± 0.90 ^c^	19.48 ± 0.91 ^b^	0.01
b*	5.05 ± 0.26 ^b^	3.90 ± 0.34 ^c^	5.42 ± 0.29 ^a^	0.01
Tenderness	103.37 ± 27.55 ^a^	86.94 ± 11.9 ^b^	66.05 ± 11.54 ^c^	0.01
Cooked Meat Yield (%)	78.77 ± 3.36	78.53 ± 4.08	65.65 ± 2.98	0.09
Drip Loss (%)	4.23 ± 1.04 ^a^	1.77 ± 0.65 ^c^	3.59 ± 0.93 b	0.02
Water Loss Rate (%)	14.89 ± 3.03 ^b^	15.35 ± 3.37 ^a^	10.38 ± 2.02 ^c^	0.01
Cooking Loss (%)	37.12 ± 4.36 ^a^	35.74 ± 8.04 ^b^	38.77 ± 2.49 ^a^	0.03

Note: each index was measured in triplicate; Duncan’s test was used for multiple comparisons, and values with different superscript letters (a–c) in the same row were significantly different (*p* < 0.05).

**Table 3:** The results of the nutritional value of the *Longissimus dorsi* for SH and their parental breeds (g/100 g (*n* = 6)).

Item	SFK	SH	HH	*p*-Value
Moisture	75.63 ± 0.55 ^a^	71.83 ± 0.42 ^c^	73.30 ± 1.23 ^b^	0.01
Ash	1.13 ± 0.15	1.20 ± 0.00	1.07 ± 0.06	0.29
Crude protein	21.39 ± 0.49	22.05 ± 0.38	20.86 ± 0.51	0.06
Crude fat	2.13 ± 0.61	2.40 ± 0.36	3.37 ± 0.98	0.16

Note: each index was measured in triplicate; Duncan’s test was used for multiple comparisons, and values with different superscript letters (a–c) in the same row were significantly different (*p* < 0.05).

**Table 4:** The results of the amino acid profiles of the *Longissimus dorsi* for SH and their parental breeds (g/100 g (*n* = 6)).

Item	SFK	SH	HH	*p*-Value
leucine ^2,5^	1.85 ± 0.04	1.87 ± 0.09	1.76 ± 0.07	0.32
isoleucine ^2,5^	1.05 ± 0.03	0.96 ± 0.09	0.91 ± 0.06	0.08
phenylalanine ^2,5^	0.93 ± 0.02	0.94 ± 0.01	0.91 ± 0.01	0.14
valine ^2,5^	1.13 ± 0.02	1.06 ± 0.02	1.06 ± 0.08	0.25
methionine ^2,5^	0.59 ± 0.03	0.58 ± 0.06	0.55 ± 0.07	0.66
lysine ^2,4^	2.02 ± 0.04	2.03 ± 0.07	1.95 ± 0.12	0.47
threonine ^2,4^	1.00 ± 0.03	1.05 ± 0.04	0.98 ± 0.07	0.23
aspartic acid ^1,3^	2.05 ± 0.04	2.10 ± 0.08	2.00 ± 0.17	0.56
tyrosine ^1^	0.78 ± 0.03	0.80 ± 0.03	0.76 ± 0.02	0.35
serine ^1,4^	0.81 ± 0.02 ^b^	0.89 ± 0.03 ^a^	0.82 ± 0.04 ^b^	0.03
glutamic acid ^1,3^	3.77 ± 0.08	3.92 ± 0.16	3.62 ± 0.17	0.10
glycine ^1,3,4^	0.95 ± 0.02 ^b^	1.03 ± 0.03 ^a^	0.95 ± 0.03 ^b^	0.01
alanine ^1,3,4^	1.31 ± 0.01 ^b^	1.34 ± 0.02 ^a^	1.22 ± 0.02 ^c^	0.01
cysteine ^1,3,4^	0.21 ± 0.06	0.21 ± 0.02	0.19 ± 0.03	0.82
histidine ^1,5^	0.72 ± 0.05	0.71 ± 0.06	0.66 ± 0.03	0.34
arginine ^1,5^	1.46 ± 0.04	1.50 ± 0.06	1.42 ± 0.08	0.38
proline ^1,4^	0.75 ± 0.08	0.82 ± 0.10	0.78 ± 0.07	0.66
essential amino acids (EAAs)	8.57 ± 0.19	8.49 ± 0.26	8.12 ± 0.53	0.34
non-essential amino acids (NEAAs)	12.81 ± 0.29 ^b^	13.32 ± 0.18 ^a^	12.42 ± 0.30 ^c^	0.02
total amino acids (TAAs)	21.38 ± 0.48	21.81 ± 0.43	20.54 ± 0.83	0.10
umami amino acids (UAAs)	9.13 ± 0.18	9.35 ± 0.33	8.70 ± 0.53	0.12
sweet amino acids (SAAs)	6.85 ± 0.13 ^b^	7.17 ± 0.05 ^a^	6.70 ± 0.11 ^c^	0.01
bitter amino acids (BAAs)	7.73 ± 0.24	7.62 ± 0.23	7.27 ± 0.46	0.22
EAAs/TAAs (%)	40.08 ± 0.32	38.93 ± 0.28	39.53 ± 0.40	0.47
EAAs/NEAAs (%)	66.90 ± 0.56	63.74 ± 0.49	65.38 ± 0.63	0.52

Note: ^1^ non-essential amino acids are marked with superscript number 1; ^2^ essential amino acids are marked with superscript number 2; ^3^ umami amino acids are marked with superscript number 3; ^4^ sweet amino acids are marked with superscript number 4; ^5^ bitter amino acids are marked with superscript number 5. Each index was measured in triplicate; Duncan’s test was used for multiple comparisons, and values with different superscript letters (a–c) in the same row were significantly different (*p* < 0.05).

**Table 5:** The results of the fatty acid profiles of the *Longissimus dorsi* for SH and their parental breeds (mg/100 g (*n* = 6)).

Fatty Acid	SFK	SH	HH	*p*-Value
Saturated fatty acids (SFAs)				
caprylic acid (C8:0)	1.06 ± 0.23 ^a^	0.44 ± 0.09 ^b^	1.10 ± 0.36 ^a^	0.01
capric acid (C10:0)	4.01 ± 2.07	2.62 ± 0.92	4.17 ± 2.15	0.07
lauric acid (C12:0)	3.89 ± 2.07	1.75 ± 0.33	3.52 ± 1.99	0.09
tridecanoic acid (C13:0)	0.86 ± 0.20	0.30 ± 0.16	0.82 ± 0.27	0.16
myristic acid (C14:0)	60.53 ± 12.34 ^b^	32.07 ± 12.46 ^b^	69.87 ± 32.38 ^a^	0.02
pentadecanoic acid (C15:0)	7.66 ± 1.28	5.33 ± 2.00	8.78 ± 3.91	0.10
palmitic acid (C16:0)	639.33 ± 95.43	445.33 ± 169.16	696.83 ± 310.70	0.13
margaric acid (C17:0)	24.35 ± 3.29	23.46 ± 10.04	27.75 ± 13.26	0.73
stearic acid (C18:0)	285.50 ± 22.59	240.25 ± 105.00	329.33 ± 167.76	0.43
arachidic acid (C20:0)	2.10 ± 0.36 ^b^	1.62 ± 0.50 ^c^	2.68 ± 0.81 ^a^	0.02
Monounsaturated fatty acids (MUFAs)				
myristoleic acid (C14:1)	2.88 ± 0.91 ^a^	1.22 ± 0.23 ^c^	3.03 ± 1.30 ^a^	0.01
palmitoleic acid (C16:1)	46.58 ± 12.18	28.47 ± 11.58	48.82 ± 19.67	0.06
oleic acid (C18:1)	889.83 ± 97.17	795.33 ± 322.57	1004.33 ± 440.15	0.54
gadoleic acid (C20:1)	4.01 ± 1.12 ^b^	2.09 ± 0.55 ^c^	5.02 ± 1.58 ^a^	0.01
erucic acid (C22:1)	80.20 ± 19.82 ^b^	112.60 ± 31.77 ^a^	22.12 ± 2.69 ^c^	0.01
nervonic acid (C24:1)	3.17 ± 2.89	3.75 ± 0.97	5.85 ± 3.11	0.35
Polyunsaturated fatty acids (PUFAs)				
linoleic acid (C18:2)	138.50 ± 22.01	98.60 ± 41.99	133.5 ± 66.31	0.31
conjugated linoleic acid (CLA) (C18:2)	3.67 ± 1.10 ^c^	6.46 ± 4.88 ^a^	5.85 ± 3.11 ^b^	0.04
γ-linolenic acid (C18:3)	1.58 ± 0.92	1.64 ± 0.70	1.47 ± 0.57	0.92
α-linolenic acid (C18:3)	4.36 ± 1.05	4.29 ± 2.16	4.45 ± 2.88	0.99
dihomo -γ-linolenic acid (C20:3)	3.98 ± 0.99 ^a^	2.17 ± 0.85 ^c^	3.56 ± 1.08 ^b^	0.02
eicosadienoic acid (C20:2)	2.19 ± 0.71	1.38 ± 0.16	2.37 ± 1.15	0.10
arachidonic acid (C20:4)	36.30 ± 6.47 ^a^	20.63 ± 5.38 ^b^	34.17 ± 10.46 ^a^	0.01
eicosapentaenoic acid (EPA) (C20:5)	2.15 ± 0.75	1.10 ± 1.12	1.83 ± 0.90	0.17
*cis*-13,16-docosadienoic acid (C22:2)	2.43 ± 0.67 ^b^	0.43 ± 0.05 ^c^	2.77 ± 0.72 ^a^	0.01
docosahexaenoic acid (DHA) (C22:6)	3.10 ± 2.89	0.78 ± 0.91	2.04 ± 1.01	0.13

Note: each index was measured in triplicate; Duncan’s test was used for multiple comparisons, and values with different superscript letters (a–c) in the same row were significantly different (*p* < 0.05).

**Table 6:** The results of the total fatty acid contents and their proportions in the *Longissimus dorsi* for SH and their parental breeds (mg/100 g (*n* = 6)).

Item	SFK	SH	HH	*p*-Value
Total Saturated Fatty Acids (SFAs)	1029.29 ± 128.65 ^b^	753.17 ± 185.32 ^c^	1144.85 ± 386.42 ^a^	0.03
Total Monounsaturated Fatty Acids (MUFAs)	1026.67 ± 115.42	943.46 ± 345.78	1089.17 ± 468.35	0.08
Total Polyunsaturated Fatty Acids (PUFAs)	198.26 ± 28.63 ^a^	137.48 ± 48.32 ^b^	192.01 ± 78.56 ^a^	0.04
Total Unsaturated Fatty Acids (TUFAs)	1224.93 ± 136.75	1080.94 ± 387.64	1281.18 ± 532.11	0.07
Total Fatty Acids (TFAs)	2254.22 ± 265.40 ^b^	1834.11 ± 572.96 ^c^	2426.03 ± 918.53 ^a^	0.03
UFAs/SFAs Ratio	1.18 ± 0.15 ^b^	1.43 ± 0.21 ^a^	1.12 ± 0.18 ^b^	0.02
n-3 PUFAs	9.61 ± 4.69 ^a^	6.17 ± 4.19 ^c^	8.32 ± 4.79 ^b^	0.04
n-6 PUFAs	186.46 ± 32.16	129.93 ± 53.85	181.32 ± 82.25	0.48
n-6/n-3 Ratio	19.40	21.06	21.79	0.60

Note: each index was measured in triplicate; Duncan’s test was used for multiple comparisons, and values with different superscript letters (a–c) in the same row were significantly different (*p* < 0.05).

**Table 7:** The qualitative results of volatile flavor compounds in the *Longissimus dorsi* for SH and their parental breeds (*n* = 6).

Compound Name	RI	RT (s)	SFK	SH	HH	*p*-Value
Esters						
ethyl-2-methyl propanoate	712.6	173.004	1314.8 ± 447.7 ^a^	1129.5 ± 551.32 ^b^	465.8 ± 299.64 ^c^	0.01
isoamyl formate	780.4	208.046	69.4 ± 27.74 ^b^	122.3 ± 71.91 ^a^	51.9 ± 20.50 ^c^	0.04
ethyl formate	516.4	114.775	299.0 ± 52.49 ^c^	593.1 ± 91.19 ^a^	459.1 ± 201.33 ^b^	0.01
methyl acetate	509.1	113.106	278.5 ± 31.11	322.8 ± 168.33	208.9 ± 54.19	0.19
propyl acetate	664.7	154.48	93.2 ± 11.88 ^c^	128.2 ± 23.89 ^b^	176.3 ± 19.97 ^a^	0.01
methyl butanoate	711.8	172.615	141.5 ± 46.39 ^a^	121.2 ± 32.38 ^b^	50.0 ± 14.57 ^c^	0.01
Alcohols						
n-heptanol	914.4	277.371	123.7 ± 55.44	135.2 ± 65.31	80.5 ± 18.87	0.18
(E)-2-hexenol	864.3	245.002	86.0 ± 15.05	78.4 ± 24.62	77.8 ± 43.60	0.87
isobutanol	604.4	136.91	306.1 ± 48.60 ^c^	533.1 ± 91.26 ^a^	430.0 ± 90.54 ^b^	0.01
3-methyl-1-butanol	694.2	164.529	159.8 ± 91.51 ^b^	306.6 ± 262.32 ^a^	61.9 ± 46.99 ^c^	0.03
Ketones						
cyclopentanone	789.6	212.392	325.9 ± 103.55 ^a^	292.7 ± 150.86 ^b^	159.2 ± 53.52 ^c^	0.04
acetoin	654.2	151.272	1266.6 ± 1110.41 ^b^	2620.3 ± 1011.51 ^a^	472.9 ± 149.86 ^c^	0.01
2-methyl-dihydro-3(H)-furanone	791.8	213.293	36.3 ± 9.34 ^a^	31.7 ± 17.60 ^b^	15.5 ± 4.11 ^c^	0.02
4-methyl-2-pentanone	699	166.72	89.6 ± 8.29 ^c^	142.5 ± 18.41 ^b^	203.2 ± 31.85 ^a^	0.01
diacetyl	568.7	127.462	134.1 ± 11.17	127.1 ± 44.36	102.1 ± 9.11	0.13
9-fluorenone	287.7	72.597	17.6 ± 8.76 ^c^	131.8 ± 68.29 ^a^	30.9 ± 4.92 ^b^	0.01
Acids						
acetic acid	627.5	143.375	309.5 ± 39.33 ^c^	405.5 ± 72.41 ^b^	505.5 ± 96.26 ^a^	0.01
Aldehydes						
3-methyl-2-butenal	715.5	174.362	190.7 ± 19.08 ^a^	152.5 ± 64.75 ^b^	86.1 ± 25.12 ^c^	0.01
3-methylbutanal	658.7	152.63	509.6 ± 45.33	530.6 ± 303.94	363.1 ± 26.29	0.24
3-methylthiopropanal	898.3	263.499	111.3 ± 69.37 ^b^	149.2 ± 75.56 ^a^	50.3 ± 19.84 ^c^	0.04
2-methyl-2-propenal	566.1	126.795	592.1 ± 176.05 ^a^	154.5 ± 26.67 ^c^	176.6 ± 81.37 ^b^	0.01
2-methylbutanal	695.5	165.126	164.7 ± 97.47 ^b^	212.0 ± 69.49 ^a^	34.4 ± 14.70 ^c^	0.01
butanal	632.9	144.933	418.7 ± 102.75	430.6 ± 97.99	314.2 ± 43.04	0.06
Thiazoles						
thiazole	717.8	175.448	485.5 ± 48.77 ^a^	445.6 ± 113.95 ^b^	348.4 ± 106.62 ^c^	0.04
Hydrocarbons						
(Z)-2-butene	436	97.706	579.2 ± 150.72 ^a^	157.1 ± 44.81 ^c^	326.2 ± 98.58 ^b^	0.01
(Z)-2-pentene	529.3	117.793	49.5 ± 8.89	63.3 ± 83.94	30.2 ± 6.78	0.52
propene	284	72.059	54.3 ± 13.22	70.0 ± 12.57	71.2 ± 16.95	0.11
1-butene	393.9	89.795	1102.6 ± 314.67	831.5 ± 720.92	1490.9 ± 496.50	0.14
fluorene, 2-methyl	285.3	72.255	36.5 ± 7.46 ^b^	70.3 ± 19.02 ^a^	38.5 ± 2.35 ^b^	0.01
Others						
1-propanethiol	625.3	142.754	87.3 ± 19.56 ^c^	126.7 ± 56.61 ^b^	243.6 ± 137.18 ^a^	0.02
2-butylfuran	894	259.865	129.1 ± 49.06 ^b^	224.5 ± 119.19 ^a^	101.3 ± 21.98 ^c^	0.03
2-ethylfuran	702.8	168.435	417.4 ± 83.10	414.1 ± 256.61	296.3 ± 47.00	0.35

Note: each parameter was measured in duplicate; Duncan’s test was used for multiple comparisons, and values with different superscript letters (a–c) in the same row were significantly different (*p* < 0.05).

## Data Availability

The original contributions presented in this study are included in the article. Further inquiries can be directed to the corresponding authors.
